# Off-Label Use of Liraglutide in the Management of a Pediatric Patient with Type 2 Diabetes Mellitus

**DOI:** 10.1155/2013/703925

**Published:** 2013-10-03

**Authors:** Sara J. Micale, Michael P. Kane, Eileen Hogan

**Affiliations:** ^1^Department of Pharmacy Practice, Albany College of Pharmacy and Health Sciences, 106 New Scotland Avenue, Albany, NY 12208, USA; ^2^The Endocrine Group, LLP, Albany, NY 12206, USA

## Abstract

Liraglutide is a glucagon-like peptide 1 (GLP-1) analog indicated for the treatment of type 2 diabetes mellitus as an adjunct to diet and exercise in adults. Liraglutide lowers blood glucose levels by stimulating insulin secretion and decreasing glucagon release in glucose-dependent manners, increases satiety, and delays gastric emptying. Liraglutide, unlike metformin and insulin, is not approved for use in the pediatric population. We report the successful off-label use of liraglutide in an obese, 16 year old Caucasian female with type 2 diabetes mellitus.

## 1. Introduction

The incidence of obesity in children is increasing, and this has been accompanied by a substantial rise in cases of Type 2 diabetes mellitus (T2DM) [1, 2]. While it was rare to see T2DM in pediatric patients thirty years ago, presently approximately 3,700 children and adolescents are diagnosed with T2DM annually [3, 4]. The highest rates of diagnosis are observed in adolescents greater than 15 years of age, diagnosis is more common in females [4], and the majority of these patients have at least one parent with diabetes mellitus [3, 5]. Despite the availability of twelve classes of agents, currently the only therapies FDA-approved for pediatric patients with T2DM are metformin and insulin. 

Liraglutide is a long-acting human glucagon-like peptide 1 (GLP-1) analog that was approved by the FDA in 2010 as an adjunct to diet and exercise to improve glycemic control in adults with T2DM [6]. It has 97% homology to native GLP-1 [6]. The amino acid substitution and a C-16 palmitic acid side chain attached via a glutamic acid spacer allow for slower drug absorption and enhance resistance to dipeptidyl peptidase-IV (DPP-4) inactivation in comparison to native GLP-1 [7]. The half-life of liraglutide is 13 hours and maximum concentrations are reached within 10–14 hours of administration making it suitable for daily dosing [6, 7]. Liraglutide increases glucose-dependent insulin release from B cells and decreases glucagon secretion from pancreatic alpha cells in glucose-dependent manners, increases satiety, and delays gastric emptying [6]. The result of these effects is improved control of postprandial and fasting glucose levels. An average weight loss of 2.5 kg in Type 2 diabetes patients with an average baseline A1C of 8.2% has been reported in adults receiving liraglutide monotherapy [6]. 

We report the first published case of off-label use of liraglutide in an obese, pediatric patient with Type 2 diabetes mellitus. In consideration of the use of this agent in a pediatric patient with T2DM and previous to writing this case report, a PubMed literature search was performed using the terms liraglutide, pediatrics, children, adolescents, and Type 2 diabetes mellitus in an attempt to identify any research or case reports discussing the use of liraglutide in pediatric patients. In addition, the manufacturer of liraglutide (Novo Nordisk) was contacted in an attempt to identify pertinent information. A poster reporting the safety, tolerability, and pharmacokinetics/pharmacodynamics of liraglutide in twenty-one pediatric T2DM patients, which was presented at the June, 2012, American Diabetes Association Annual meeting, was the only information identified; however, drug efficacy was not a primary endpoint [8]. This is the first case report demonstrating the efficacy of liraglutide in the pediatric population.

## 2. Case Report

An obese, 16-year-old, Caucasian female with a 4-year history of T2DM presented to our endocrinology practice for a follow-up visit on October 26th, 2012. She was diagnosed with diabetes at the age of 12 and has a significant family history for Type 2 diabetes and obesity. Her medical history is significant for T2DM complicated by microalbuminuria, depression, and type IV hyperlipidemia. She was referred to the practice for management of T2DM at the age of 14 with a hemoglobin A1c (HbA1c) of 11.47. Her HbA1c has not been below 11% in the past 2 years, including an A1C of 13.4% in July 2012. 

In September 2012, the patient presented with her mother for a follow-up visit. The patient stated she tries to control her blood sugar and weight but gets frustrated and reverts to poor habits. Her parents are not helpful in assisting her in managing her medical problems or establishing healthy eating habits. Her weight at this visit was 244 lbs. With a height of 66 inches; her BMI was 39.4 kg/m^2^. Prior to this visit, she managed her diabetes with insulin glargine 75 units subcutaneously in the morning and 25 units at night, metformin ER 2000 mg once daily, and insulin aspart at mealtime using a coverage scale, as the patient and parents were reluctant to use scheduled mealtime insulin doses. Her HbA1c was 11.19% at this visit. After careful consideration and dutiful explanation to the patient and her mother (including the fact that no other medication is currently approved for use in pediatric patients), consent was given, and liraglutide 0.6 mg subcutaneously daily was added to the patient's diabetes regimen. The patient was instructed to titrate the dose of liraglutide up to 1.8 mg daily over a three-week period. 

On October 26th, 2012, the patient returned to the office for a 1-month follow-up visit since the initiation of liraglutide; she was currently using 1.8 mg subcutaneously daily. She reported no adverse effects from the medication and no symptoms of hyperglycemia or hypoglycemia. During this time her HbA1c decreased by 1.53% to 9.66%. In addition, her total daily basal insulin requirement decreased 20% from baseline and she rarely needed insulin aspart for mealtime coverage of glucose excursions. Weight was 11 pounds higher than at her previous visit. Nutritional counseling was again addressed. On November 27th, 2012, the patient returned to the office for a follow-up visit two months after initiation of liraglutide. The patient stated that she has not experienced any symptoms of hypoglycemia or hyperglycemia. Her HbA1c decreased an additional 0.82% to 8.84%, representing a total A1C reduction of 2.35%. The patient's diabetes regimen included liraglutide 1.8 mg subcutaneously prior to dinner, insulin glargine 60 units subcutaneously at bedtime, and metformin ER 2000 mg with the evening meal. Since the initiation of liraglutide the patient's basal insulin requirement has decreased by 40%, and she no longer requires the use of mealtime insulin. Weight at this visit was increased, however, by 1.8 pounds (up to 256.8 pounds and a BMI of 41.4 kg/m^2^). 

## 3. Discussion

We report a rapid glycemic response to the off-label use of liraglutide in an obese, pediatric patient with Type 2 diabetes mellitus. The incidences of obesity and Type 2 diabetes mellitus in the pediatric population are rising rapidly and are linked to the sedentary lifestyle demonstrated by many American children [2, 9]. The rates of obesity in adults and children dramatically increased through the 1980s and 1990s, with greater than 35% of adults and 16.9% of US children and adolescents aged 2–19 years old classified as obese [10, 11]. Obesity and diabetes mellitus can lead to concurrent comorbid conditions such as hypertension, hyperlipidemia, and microvascular and macrovascular complications [1, 5, 10]. Although much information exists regarding the management of T2DM in adults, data and recommendations are lacking regarding T2DM management in the pediatric population [3, 5]. It has been reported that the rate of microvascular complications may be increased in children diagnosed with T2DM at an early age compared to adults diagnosed at much older ages [5]. Indeed, our patient has already been diagnosed with microalbuminuria. Appropriate therapy for children and adolescents with T2DM is essential to prevent or potentially delay the onset of complications due to poor glycemic control [5, 12].

Currently there are only two FDA approved therapies for pediatric patients with Type 2 diabetes mellitus: metformin and insulin [5, 12]. Use of insulin is associated with significant weight gain and risk of hypoglycemia, and therefore may not be the ideal agent for first-line therapy in the pediatric population [9] especially since most pediatric patients diagnosed with Type 2 diabetes mellitus are already overweight or obese, and the use of insulin may only further exacerbate that problem. Metformin has demonstrated improvement in fasting plasma glucose levels and HbA1c in pediatric patients with doses up to 2,000 mg daily [9]. The TODAY study evaluated the use of metformin monotherapy, metformin with rosiglitazone, or metformin with lifestyle education in 699 adolescents between 10 and 17 years old. The authors reported that metformin and rosiglitazone in combination was superior to the other treatment arms for glycemic control in these patients. This study supports the notion that adequate control (and potentially prevention of diabetes-related complications) may not be achieved with monotherapy in adolescents with T2DM [2]. 

Many providers do not feel comfortable prescribing medications off label, especially in the pediatric population. Except for metformin and insulin, there is a medical necessity for formal evaluation of all other currently available diabetes medications regarding their safety and efficacy in the pediatric T2DM population. Lack of dosing information regarding the use of these agents in pediatric patients can result in adverse outcomes from under dosing and not achieving full therapeutic effects, over dosing and increasing drug toxicity, or patients not receiving any medication because of safety concerns and lack of efficacy data [12]. The Pediatric Exclusivity “Rule” under Section  505A of the Federal Food, Drug, and Cosmetic Act was created to aid in the approval of medications in the pediatric population [12–14], allowing the manufacturer to evaluate the safety and efficacy of drugs and biologic products in the pediatric population. In return, the manufacturer receives an additional six months of patent exclusivity for conducting these studies [12, 13]. Another potential benefit of the Pediatric Exclusivity Rule is the potential for the development of new product formulations that are more suitable for children as the FDA is authorized to require manufacturers to produce the drug in a suitable formulation for the pediatric population in exchange for an extended patent [14]. 

This is the first case report discussing the usage of liraglutide in the pediatric Type 2 diabetes mellitus population. Klein et al. presented the safety, tolerability, and pharmacokinetics/pharmacodynamics data of liraglutide in 21 pediatric patients with T2DM at the June, 2012, ADA annual meeting which did not include drug efficacy as a primary study endpoint [8]. Eligibility requirements for that study included children between the ages of 10–17 years with T2DM, initial treatment with metformin or lifestyle changes alone, a HgA1c between 6.5–11%, a fasting plasma glucose (FPG) level of 110–240 mg/dL, and a BMI > 85 percentile based on age and gender. The authors reported that no serious adverse events or severe hypoglycemic episodes occurred in any patient and that no clinically significant differences in safety or tolerability were reported. From a pharmacodynamic perspective, there was a significant reduction in HbA1c with liraglutide when compared to placebo (*P* = 0.0007); however, no differences were seen from change in baseline in FPG or body weight between comparators [8]. An expected paper detailing these results should provide insight regarding the use of liraglutide in pediatric Type 2 patients and may result in the initiation of a large, phase 4 study assessing the safety and efficacy of liraglutide in pediatric patients with T2DM (personal communication, Novo Nordisk). The patient in our case report demonstrated a positive response to liraglutide with a decrease in HgA1c by 2.35% after just two months of therapy.

Liraglutide use is typically associated with weight loss in the adult T2DM population [6]. In contrast, after two months of therapy, our patient actually gained 12.8 pounds, or 5.2% of additional body weight. While this may be due to a decrease of glucosuria, appropriate nutritional counseling was reinforced with the patient. Interestingly, Klein et al. did not report weight loss in their poster presentation [8]. Further phase 4 studies are needed to determine any effect that liraglutide may have on weight in the pediatric population. 

## 4. Conclusion

To our knowledge, this is the first published case report demonstrating the efficacy of liraglutide in a pediatric patient with Type 2 diabetes mellitus. The addition of liraglutide allowed this patient to safely decrease her HbA1c by 2.35% with just two months of therapy without reported adverse events. Pediatric Type 2 patients currently have few options to control their diabetes. This is especially concerning because of the fact that Type 2 diabetes is becoming more prevalent in children and adolescents. Currently, an industry-sponsored phase 4 trial is actively recruiting patients to assess the safety and efficacy of liraglutide in the T2DM pediatric population. Additional prospective, long-term, randomized trials are desperately needed to evaluate the safety and efficacy of other available diabetic medications for the optimal management of pediatric T2DM. 
